# Scientists and Civil Society Must Move Together toward a New Science

**DOI:** 10.3389/fpubh.2016.00096

**Published:** 2016-05-11

**Authors:** Christian Vélot

**Affiliations:** ^1^Laboratoire VEAC, Faculté des Sciences, Univ. Paris-Sud, Université Paris-Saclay, Orsay, France; ^2^Pôle Risques MRSH-CNRS, Université de Caen, Caen, France; ^3^CRIIGEN, Paris, France; ^4^Fondation Sciences Citoyennes, Paris, France

**Keywords:** co-construction of knowledge, participatory research, risk assessment, GMOs, pesticides, civil society

In the current context of research and innovation that are increasingly driven by short-term industrial interests, science and technology require thorough social, political, ethical, and legal changes leading to better democratic control. A huge gap has opened between citizens and scientists, with the latter sometimes inspiring more mistrust than trust. Major health and environmental scandals of past years (for example, asbestos, Bovine Spongiform Encephalopathy, PCBs, and nuclear disasters) may be related to this situation.

To restore the links between science, policy makers, and civil society is a difficult task with many challenges. This involves (a) substituting a research approach strictly entrusted to the scientific community, with approaches based on a willingness to access and respect various forms of knowledge; (b) taking into account, at a very early stage in public research policy, the societal challenges of science and the tools for its democratic orientation; (c) expanding access to scientific knowledge in society, allowing those that are often wrongly called “ignorant” to interact with researchers in a balanced dialog and a co-construction of knowledge. How is it conceivable, for instance, to develop an agricultural research project without a close exchange and collaboration with those people who invented agriculture – not the researchers, or even the agronomists, but farmers? Moreover, in a knowledge society, in which innovation does not necessarily mean “progress,” citizens may be especially willing to participate in choosing scientific and technological orientations.

Such a task implies in particular the setting up of systems enabling civil society to access opportunities to develop scientific knowledge, as well as for innovation and expertise (1). Participatory research, which is joint research work with equal partnerships between non-profit organizations from civil society or groups of citizens and academic researchers (from universities or major research organizations), is an integral part of this process of democratization of science. Several public programs successfully promote participatory research. Examples include the Canadian program of *Community-University Research Alliances* (ARUC)^1^; several regional research programs in France, such as *Partnerships between Institutions and Citizens for Research and Innovation* (PICRI),^2^ set up by the Region Ile-de-France under the leadership of the *Fondation Sciences Citoyennes* organization^3^; and the *Social Appropriation of Sciences* (ASOSC),^4^ developed by the Brittany Region.

A project resulting from collaboration between researchers and actors of civil society often addresses a societal issue. Thus, participatory research involves mainly applied research projects and projects that fall within the field of expertise (health, environmental, ethical, etc.). For basic research, i.e., research that is conducted solely for the sake of increasing human knowledge, such collaborations are more difficult to consider, since this research generally falls into skills that are specifically those of scientists. However, citizens can participate in some basic research projects, by collecting data on a large scale (for example, in the field of biodiversity). In this case, participants train themselves with the files and protocols that are given beforehand. Besides, citizens could be consulted about the fundamental questions that they are concerned with and that they would like to be addressed by scientists.

The requirement for openness of science to civil society is particularly striking in the area of technologies and the products of technology. The phenomenon of lucrative research driven by industrial interests that require rapid returns on investment leads to negative consequences in terms the quality and transparency of health and environmental assessment. The time required to conduct these assessments with proper rigor is not compatible with the urgency of patents and profits, and commercial confidentiality is used to justify the failure to communicate raw data from regulatory tests.

Many civil society organizations have emerged to oppose the possible assessment deficiencies of new products placed on the market. These organizations play an important role at the interface between the assessment authorities and civil society as a whole, not as mediators, but as shields designed to protect citizens from potential hazards resulting from inadequate assessments. Participatory research projects provide an opportunity for civil society organizations to intervene as interlocutors and collaborators with scientists who are engaged in research on assessment issues. These organizations are then able to relay the results to the general public and decision makers and develop arguments for possible revision of assessment regulations by public authorities.

An example of this was provided by the international conference on “Assessment and Regulation of Genetically Modified Organisms (GMOs) and Pesticides,”^5^ held in France at the Orsay Scientific Centre (University of Paris-Sud) on 12 and 13 November 2015.

The originality of this symposium was the fact that it was open to civil society (with French/English simultaneous translation), organized as part of a participatory research (PICRI project, funded by the Île-de-France Region), and managed collaboratively between University Paris-Sud and two associations: *Générations Futures*^6^ and the *Committee for Research and Independent Information on Genetic Engineering (CRIIGEN)*.^7^ This project^8^ focuses on the study of the “substantial equivalence” principle (i.e., close nutritional and element similarity between two crop-derived foods), which has been used as the basis to allow the commercial approval of all agricultural GMOs cultivated across the world. This concept, adopted by the Organization for Economic Co-operation and Development (OECD) in 1993 and endorsed by the Food and Agriculture Organization (FAO) and World Health Organization (WHO) in 1996, is registered in the Food and Drug Administration (FDA) regulation (*Part IX: Foods derived from New Plant Varieties*) and was used to claim that GM crops are as safe and nutritious as currently consumed plan-derived foods (2). Since this concept applies at the chain end (i.e., to the food from these plants), it should consider the context of the growing crops. Precisely, in the case of herbicide-tolerant GM crops, this context is not the same as for their conventional counterparts since the former are sprayed with the herbicide. Surprisingly, during substantial equivalence studies, either the tested plants are not sprayed with the recommended herbicide (3) or the herbicide residues are not measured anyway (4).

The international dimension of the conference was not only due to the panel of speakers but also to those who attended. Among the 140 participants, many came from different countries (not only in Europe but also in America and Africa).

According to the spirit of participatory research, this conference allowed the creation of a bridge between academic research and the “scientific third sector” (citizens, associations, NGOs, policy makers), with presentations of experimental scientific data, made accessible to the general public, as well as round tables bringing together civil society stakeholders. Both were followed by long interactions with the public. This spirit was also the reason why we chose to organize this conference at the Orsay Scientific Centre: because companies are allowed to sit on university boards of directors, it was important for my colleagues and myself, concerned about the democratization of science, to offer the opportunity for citizens to penetrate inside the walls of the university.

Together with results from Brazilian (5) and Norwegian (6) research groups, some of the results of the PICRI project (7) questioned the relevance of the substantial equivalence principle, especially when used to approve the commercialization of herbicide-tolerant GM crops. The second scientific session offered a state-of-the-art review of experimental data showing the insufficiencies of regulatory toxicity tests of GMOs and pesticides (8, 9). It was emphasized that the duration of feeding trials is insufficient to detect potential chronic (long-term) toxic effects and that contamination of laboratory rodent diets by toxic environmental pollutants is a confounding factor in regulatory tests. Some of the results presented at this conference showed that commercial formulations of pesticides are always more toxic than their so-called “active principles.” Yet, the latter are tested alone to calculate safety thresholds, even though they are never used in isolation, but always mixed with toxic adjuvants. Last but not least, it was explained that regulatory tests are unable to detect endocrine disrupting effects, a common toxic mechanism shared by many pesticides. During the third scientific session, the panel of speakers showed the detrimental effects of pesticides and GMOs on soil ecosystems, for example, on rhizosphere microflora and earthworms (10), and on food microorganisms (11).

Round tables allowed exchanges on various models of participatory research and highlighted the need to involve civil society in research programs and in the choice of major research directions. The regulation of pesticides and GMOs (including those resulting from the use of new genetic engineering technologies other than transgenesis) at national and European levels was also widely discussed. There was a particular emphasis on the questions of data transparency, conflicts of interests in assessment committees, and the responsibility of experts and policy makers. Finally, a farmer, an agronomist, a physician, and a company manager producing flour and animal feed explained and exchanged their views on the question of agricultural sustainability and food choices in the future.

These 2 days of exchanges, where multiple participants from different backgrounds contributed to a unified collective intelligence, were particularly rich and intense. They confirmed the need to open up science to the public, not only to share results but also to build on each other’s knowledge.

To this end, and in line with proposals made by *Fondation Sciences Citoyennes*,^9^ the following arrangements must be implemented:

(a) integrating participatory research programs in all public research policies;
(b) taking into consideration the value of the participation of civil society (non-profit) to research;
(c) setting up evaluation criteria for researchers involved in participatory research projects;
(d) supporting the mobility of researchers toward civil society organizations;
(e) expanding communication on participatory research to researchers, students, and civil society.

There are professional scientists – the researchers – and professional politicians, including elected officials. But the scientific approach, like political action, belongs to everyone. It is time to move toward a new science, considering the “substantial equivalence” between professional scientists and citizens.

## Author Contributions

CV is the scientific coordinator of the international conference and of the participatory research project reported in this article.

## Conflict of Interest Statement

The opinions and conclusions in this commentary are those of the author and do not necessarily represent the official position of the institutions with which he is affiliated: University Paris-Sud, Pôle Risques, MRSH, CNRS, and University of Caen.

## Footnotes

^1^http://www.sshrc-crsh.gc.ca/funding-financement/programs-programmes/cura-aruc-fra.aspx

^2^http://www.iledefrance.fr/competence/picri

^3^http://sciencescitoyennes.org

^4^http://xnet1.region-bretagne.fr/Recherche

^5^http://picriogm.weebly.com/colloque.html

^6^http://www.generations-futures.fr

^7^http://www.criigen.org

^8^http://www.picri-ogm.fr

^9^http://sciencescitoyennes.org/rubrique/tiers-secteur-de-la-connaissance/recherche-participative/
